# Therapeutic hypercapnia after cardiac arrest: still promising or time to move on?

**DOI:** 10.1186/s40635-026-00974-6

**Published:** 2026-09-18

**Authors:** Wiput Laosuksri, Borwon Wittayachamnankul, Siriporn C. Chattipakorn, Nipon Chattipakorn

**Affiliations:** 1 Department of Emergency Medicine, Faculty of Medicine, Chiang Mai University, Chiang Mai, 50200 Thailand; 2 Cardiac Electrophysiology Research and Training Center, Faculty of Medicine, Chiang Mai University, Chiang Mai, 50200 Thailand; 3 Center of Excellence in Cardiac Electrophysiology Research, Chiang Mai University, Chiang Mai, 50200 Thailand; 4 Department of Oral Biology and Diagnostic Sciences, Faculty of Dentistry, Chiang Mai University, Chiang Mai, 50200 Thailand; 5 Cardiac Electrophysiology Unit, Department of Physiology, Faculty of Medicine, Chiang Mai University, Chiang Mai, 50200 Thailand; 6 The Academy of Science, The Royal Society of Thailand, Bangkok, Thailand

**Keywords:** Hypercapnia, Neurological outcome, Normocapnia, Post-cardiac arrest, Survival

## Abstract

Post-cardiac arrest care remains challenging, with persistently poor neurological and survival outcomes. Accordingly, novel adjunctive interventions are needed. While maintaining normocapnia is the current standard, hypercapnia has been proposed as a potential intervention. However, its role and potential benefits remain unclear. This comprehensive review summarizes current evidence on the use of hypercapnia in post-cardiac arrest care, focusing on neurological, cardiovascular, and survival outcomes, underlying mechanisms, and directions for future clinical investigations. Observational studies have sparked interest in the potential benefits of hypercapnia during the post-cardiac arrest period, including in specific populations. Methodological variations and conflicting findings from earlier studies have prompted the conduct of controlled trials and animal experiments to clarify its effects. Mild hypercapnia appears to be a practical and clinically safe approach, improving cerebral oxygenation without major adverse events. However, no improvement in long-term neurological recovery or survival has been demonstrated. Moreover, the effects on the cardiovascular and other organ systems, as well as mechanisms at the cellular level, remain inconclusive. Although mild hypercapnia may be considered part of a comprehensive post-cardiac arrest care strategy, further studies are needed to define its optimal application, potential synergy with other interventions, the populations most likely to benefit, and its underlying mechanisms.

## Introduction

Post-cardiac arrest (PCA) syndrome, primarily resulting from ischemia/reperfusion injury, occurs after successful resuscitation [1]. Out-of-hospital cardiac arrest (OHCA) achieves return of spontaneous circulation (ROSC) in about 25% of cases, with 7–10% surviving to hospital discharge [2, 3]. Around 10% of survivors have poor neurological outcomes [2, 3]. In-hospital cardiac arrest (IHCA) shows higher survival, largely due to earlier recognition and intervention [2, 3].

PCA care primarily aims to reduce secondary brain injury, the leading cause of death and disability, by managing underlying causes and maintaining normoxia, normocapnia, stable blood pressure, and appropriate temperature control [4, 5]. American guidelines support a target temperature of 32–37.5 °C, whereas European guidelines emphasize active fever prevention below 37.5 °C [4, 5]. PCA care must also address systemic inflammation and myocardial injury [6]. Biomarkers such as neuron-specific enolase (NSE), S100 calcium-binding protein B (S100B), and serum-Tau, alongside functional outcome measures such as the Cerebral Performance Category (CPC), Glasgow Outcome Scale (GOS), and the modified Rankin Scale (mRS), can assist in early prognostication [4, 7]. PCA management continues to evolve through ongoing research aimed at improving outcomes.

Arterial carbon dioxide tension (PaCO₂) reflects the adequacy of ventilation and influences cerebral physiology. When PaCO₂ drops to hypocapnic levels, cerebral vessels constrict, which reduces cerebral blood flow (CBF) and can worsen ischemia/reperfusion injury [8]. Conversely, hypercapnia causes these vessels to dilate and increases CBF. However, this can also raise intracranial pressure (ICP) and lower cerebral perfusion pressure (CPP) [8]. In patients whose autoregulation is impaired, a common situation after global brain ischemia, the rise in ICP can become more pronounced and carry greater risks [9].

Carefully controlled hypercapnia may confer potential benefits [10–12]. In rats with cerebral ischemia not related to cardiac arrest, moderate hypercapnia (PaCO₂ between 60 and 100 mmHg) improved CBF without a significant rise in ICP [13]. This effect helped limit brain injury after transient global ischemia and provides evidence for its protective role. A clinical study also reported that PCA patients with higher PaCO₂ levels were more likely to be discharged home, raising interest in whether hypercapnia could also protect the brain in humans [11]. Beyond its vascular effects, experimental studies suggest that hypercapnia may also attenuate oxidative brain injury and excitotoxic signaling by reducing excitatory neurotransmission and enhancing inhibitory neurotransmission [14, 15].

This comprehensive review aims to summarize current evidence on the use of hypercapnia in PCA care, focusing on its association with neurological outcomes and survival. It also explores possible underlying mechanisms to clarify its clinical relevance and potentially to guide future research on post-resuscitation strategies. In this review, several shared characteristics were identified among the included clinical and in vivo studies. All studies directly compared hypercapnia (HC) with normocapnia (NC) in relation to outcomes, and the results presented here are based on these comparative analyses. The study populations comprised comatose, mechanically ventilated patients after ROSC from non-traumatic cardiac arrest.

## Methods

This article was designed as a narrative review using a structured approach to the literature search and study selection. No predefined protocol was developed or registered, and PRISMA reporting was not applied. PubMed was searched from inception to October 2025 using the following Boolean search strategy: “hypercapnia AND (cardiac arrest OR heart arrest OR CPR OR cardiopulmonary resuscitation).” English-language original clinical studies and relevant in vivo experimental studies were included if they directly compared hypercapnia or higher PaCO₂ targets with normocapnia after cardiac arrest and reported neurological, survival, cardiovascular, physiological, biomarker, or mechanistic outcomes.

Records were initially screened for relevance by one author, and uncertainties regarding study eligibility were resolved through discussion with two senior authors. The final synthesis included 35 original articles: 31 identified through the database search and 4 through reference checking. No formal quality appraisal was undertaken. The findings were synthesized narratively, taking the key limitations of the included studies into account. The use of a single database and the absence of formal systematic-review methodology were acknowledged limitations of this review.

### Neurological and survival outcomes associated with hypercapnia in post-cardiac arrest adult patients: reports from the out-of-hospital cardiac arrest model

PCA patients from OHCA generally have a worse prognosis due to delayed detection and treatment [16]. It has been shown that both mean and time-weighted exposure to HC were associated with improved CPC scores at 12 months, with good neurological outcome defined as CPC 1–2 [12]. This multicenter study used the duration of exposure to HC as a grouping variable in the analysis. It also evaluated oxygen levels and found that patients with HC combined with mild hyperoxia had the highest rates of favorable neurological recovery.

In contrast, a prospective study examining any HC exposure within the first 24 h after ROSC found no association between HC and CPC outcome [17]. Furthermore, a retrospective study demonstrated that both mean and time-weighted exposure to HC were not associated with epileptiform electroencephalography (EEG) activity or survival [18]. Moreover, two retrospective studies reported an association between HC and worse outcomes [19, 20]. In these studies, HC defined by the first or mean PaCO₂ within 24 h was associated with lower survival and poorer CPC and mRS outcomes (4–6 as poor outcome) [19, 20].

When focusing on HC levels, mild HC (above 45 – 55 mmHg) showed no association with neurological recovery or changes in serum-Tau [21]. In addition, a multicenter study that examined both functional and survival outcomes reported that HC exposure, assessed using PaCO₂ measurements at ICU admission and 24 h after ROSC and categorized as mild or severe, was not associated with survival [22].

PaCO₂ has been evaluated as one of several factors associated with survival in OHCA patients. A large multicenter cohort study analyzing PaCO₂ and PaO₂ at the lowest PaO₂/FiO₂ ratio within 24 h after admission found no significant association between HC and survival [23]. Similar findings were observed in a single-center study that analyzed the first PaCO_2_ after hospital admission, which also found no association between HC and survival [24]. Moreover, a multicenter study reported that HC detected at any time point within the first 24 h after hospital arrival was associated with increased hospital mortality [25]. However, in a multicenter cohort study using values at the lowest PaO₂/FiO₂ ratio within 24 h, it was found that HC, classified as mild (46–50 mmHg) and severe (> 55 mmHg), was associated with improved survival compared with NC or moderate HC [26], as summarized in Table 1.

**Table 1:** Neurological and survival outcomes associated with hypercapnia after adult cardiac arrest: observational evidence

**Study type/** **reference**	**Population**	**TTM** **(**^**๐**^**C)**	**Number of** **participants**		**Hypercapnia**		**Major findings**		Interpretation*	**Key limitations**
			**HC**	**NC**	**Assessment definition**	**PaCO**_**2**_** level** **(mmHg)**	**Neurological outcomes**	**Survival**		
Prospective, multicenter/ [12]	OHCA	Yes (N/A)	N/A	N/A	Mean 24-h PaCO₂ and time-weighted exposure	> 45	↑ favorable CPC at 12 mo	N/A	Hypercapnia was associated with improved neurological outcomes	PaCO₂ assessed after ICU admission; Time-weighted exposure complicated direct HC/NC group comparison
Prospective, multicenter/ [17]	OHCA	Yes (33 or 36)	670	591	Any HC exposure within 24 h after ROSC	> 50	↔ favorable CPC at hospital discharge, 6 mo	N/A	There was no association between hypercapnia and neurological outcomes	Exact exposure timing and duration unavailable
Prospective, multicenter, substudy of the TTM Trial/ [21]	OHCA	Yes (33 or 36)	685	39	Highest PaCO₂ from admission to 36 h	> 45	↔ favorable CPC at 6 mo ↔ peak serum-Tau at 48 and 72 h no interaction with TTM	N/A	Hypercapnia showed no association with neurological outcomes or interaction with targeted temperature management	PaCO₂ assessed after hospital admission
			Mild 121	675	time-weighted mean PaCO₂ from admission to 36 h	Mild > 45 – 55	↔ favorable CPC at 6 mo ↔ peak serum-Tau at 48 and 72 h no interaction with TTM	N/A		
Prospective, multicenter/ [23]	OHCA	N/A	1834	2288	PaCO₂ at the lowest PaO₂/FiO₂ within 24 h	> 45	N/A	↔ at hospital discharge with/ without adjustment for PaO_2_	Hypercapnia at the lowest PaO_2_/FiO_2_ ratio showed no association with survival	PaCO₂ assessed after ICU admission; single oxygenation-indexed measurement; neurological outcomes not assessed
Prospective, multicenter/ [25]	OHCA	N/A	4681	N/A	Any HC exposure within 24 h after hospital arrival	> 50	N/A	↓ at hospital discharge	Hypercapnia was associated with decreased survival	PaCO₂ assessed after hospital arrival; exact exposure duration unavailable; neurological outcomes not assessed
Prospective, multicenter/ [31]	Mixed IHCA&OHCA	Yes (N/A)	N/A	N/A	Mean PaCO₂ during the first 6 h after ROSC	Mild- moderate 46—68	↑ favorable mRS at hospital discharge	N/A	Mild to moderate hypercapnia was associated with a higher predicted probability of favorable neurological outcome	PaCO₂ exposure limited to the first 6 h after ROSC; no direct HC/NC group comparison
						Severe > 68	↓ favorable mRS at hospital discharge	N/A		
Prospective, single-center/ [32]	IHCA	N/A	Mild 62	151	Mean PaCO₂ within 24 h after ROSC	Mild 46 – 55	↔ favorable GOS at hospital discharge	↓ at hospital discharge	Hypercapnia was associated with lower survival rates, with severe hypercapnia linked to worse neurological outcomes, while mild hypercapnia showed no association	Mean PaCO₂ might obscure fluctuations and transitions between hypo- and hypercapnia; IHCA-only population
			Severe 51	151		Severe > 55	↓ favorable GOS at hospital discharge	↓ at hospital discharge		
Prospective, single-center/ [29]	Mixed IHCA&OHCA	Yes (N/A)	63	60	Any PaCO₂ exposure within 24 h after ROSC	≥ 50	↓ favorable CPC at hospital discharge	N/A	Hypercapnia was associated with worse neurological outcomes	Exact exposure timing and duration unavailable; heterogeneous number of PaCO₂ measurements
Prospective, single-center/ [30]	Mixed IHCA&OHCA	Yes (N/A)	17	47	Initial PaCO₂ after initiation of post-ROSC ventilator settings	≥ 50	↓ favorable CPC at hospital discharge	N/A	Hypercapnia was associated with worse neurological outcomes	Single PaCO₂ measurement; median 120-min delay from initiation of ventilation to ABG
Retrospective, multicenter/ [19]	OHCA	Yes (N/A)	83	332	Mean PaCO₂ within 24 h	> 45	↓ favorable CPC at discharge	↓ at hospital discharge and 12 mo	Hypercapnia was associated with lower survival and worse neurological outcomes	PaCO₂ assessed after ICU admission
Retrospective, multicenter, sub-study of the ALPS Trial/ [20]	OHCA	N/A	746	-	First PaCO₂ after hospital admission	> 45	↓ favorable mRS at hospital discharge	↓ at hospital discharge	The first recording of hypercapnia after admission was associated with decreased survival and unfavorable neurological outcomes, while the PaCO₂ reduction rate was lower in survivors	HC-only cohort without an NC comparator; PaCO₂ assessed after hospital admission
					PaCO₂ reduction rate between the first and second recordings		↔ favorable mRS at hospital discharge	↓ at hospital discharge		
Retrospective, multicenter/ [22]	OHCA	Yes (N/A)	Mild 98	96	PaCO₂ exposure based on two measurements: at ICU admission and 24 h after ROSC	Mild 46 – 55	↔ favorable CPC at 1 mo	↔ at 1 mo	No association was found between mild or severe hypercapnia and survival. However, severe hypercapnia was associated with worse neurological outcomes	Only two PaCO₂ measurements were used to define exposure
			Severe 88			Severe > 55	↓ favorable CPC at 1 mo	↔ at 1 mo		
Retrospective, multicenter/ [26]	OHCA	Yes (N/A)	Mild 3444	9910	PaCO₂ at the lowest PaO₂ within 24 h after ICU admission	Mild 46—50	N/A	↑ at hospital discharge	Mild and severe hypercapnia at the time of the lowest PaO_2_ were associated with higher survival	PaCO₂ indexed to the lowest PaO₂ rather than overall CO₂ exposure; PaCO₂ assessed after ICU admission; single oxygen-indexed ABG measurement; neurological outcomes not assessed; residual confounding
			Moderate 1958			Moderate 51—55	N/A	↔ at hospital discharge		
			Severe 2759			Severe > 55	N/A	↑ at hospital discharge		
Retrospective, multicenter/ [11]	Mixed OHCA/IHCA	Yes (N/A)	6,827	6,705	PaCO₂ at lowest PaO₂ within 24 h	> 45	↑ Discharge home among survivors	↔ at hospital discharge	Hypercapnia at the lowest PaO₂ was associated with discharge home but not survival	PaCO₂ indexed to the lowest PaO₂ rather than overall CO₂ exposure; PaCO₂ assessed after ICU admission; discharge destination used as a surrogate neurological outcome
Retrospective, multicenter/ [33]	Mixed IHCA&OHCA	Yes (N/A)	Mild 472	1088	Time-weighted mean PaCO₂ during the first 24 h after ICU admission	Mild 46 – 55	N/A	↔ at hospital discharge	Mild hypercapnia was not associated with survival at time of hospital discharge; however, severe hypercapnia was linked to lower survival	Cardiac arrest characteristics unavailable; PaCO₂ assessed after ICU admission; neurological outcomes not assessed
			Severe 290	1088		Severe > 55	N/A	↓ at hospital discharge		
Retrospective, multicenter/ [34]	N/A	N/A	308 (with normal pH)	1931	PaCO₂ and pH from the ABG producing the highest APACHE III subscore within 24 h after ICU admission	> 45 (with normal pH)	N/A	↔ at hospital discharge	Hypercapnic acidosis was associated with reduced survival at time of discharge, while compensated hypercapnia showed no association with survival	Study-specific PaCO₂ assessment limits comparability with other studies; cardiac arrest characteristics unavailable; neurological outcomes not assessed
			9519 (with acidosis)	1931		> 45 (with acidosis)	N/A	↓ at hospital discharge		
Retrospective, Single-center/ [18]	OHCA	Yes (33)	N/A	N/A	Percentage of time exposed to HC within 48 h	> 45	↔ epileptiform EEG activity	↔ at hospital discharge	No association was found between hypercapnia and survival or seizure events	PaCO₂ assessed after ICU admission; EEG performed only on clinical indication
Retrospective, single-center/ [24]	OHCA	N/A	N/A	N/A	First PaCO₂ within 1 h after hospital admission	> 50	N/A	↔ within 5 days	There was no association between hypercapnia and survival	HC/NC group sizes not reported; single PaCO₂ measurement after hospital admission; no neurological outcome
Retrospective, single-center/ [28]	Mixed OHCA/IHCA	Yes (33)	17	152	Mean PaCO₂ from ROSC to end of rewarming	> 45	↔ favorable CPC at hospital discharge	↔ at hospital discharge	Hypercapnia showed no association with neurological outcomes or survival	Small HC group; TTM-only cohort; timing effects not assessed

*Interpretation compares findings in the hypercapnia group with the normocapnia group. ↑ indicates an increase or higher rate of the specified outcome; ↓ indicates a decrease or lower rate of the specified outcome; ↔ indicates no significant association or difference

ABG, arterial blood gas; CPC, Cerebral Performance Category; EEG, electroencephalography; GOS, Glasgow Outcome Scale; HC, hypercapnia; IHCA, in-hospital cardiac arrest; mRS, modified Rankin Scale; NC, normocapnia; OHCA, out-of-hospital cardiac arrest; PaCO₂, partial pressure of arterial carbon dioxide; PaO₂, partial pressure of arterial oxygen; PaO₂/FiO₂, ratio of arterial oxygen partial pressure to fractional inspired oxygen; ROSC, return of spontaneous circulation; TTM, targeted temperature management.

Findings remain inconsistent despite similar baseline characteristics, with most patients aged 59–67 years, treated with targeted temperature management (TTM), and presenting with shockable rhythms, consistent with typical OHCA populations [27]. One key reason for this inconsistency is the substantial variation in the definitions of HC, including thresholds of PaCO₂ above 45 or 50 mmHg, the timing of arterial blood gas sampling, and the criteria used for classification [12, 17–26]. Similar heterogeneity has also been observed in studies including IHCA or mixed IHCA/OHCA populations. The potential influence of HC level, PaCO₂ assessment method, and exposure timing on clinical outcomes is synthesized across these populations in the following section.

### Neurological and survival outcomes associated with hypercapnia in post-cardiac arrest adult patients: reports from in- and out-of-hospital cardiac arrest models

Studies including IHCA, alone or combined with OHCA, are summarized in Table 1. Schneider et al. were among the first to report an association between HC and better neurological outcomes, sparking interest in this topic [11]. This large multicenter study found that HC was independently associated with a higher likelihood of discharge home among survivors, although overall survival did not differ from NC [11]. Notably, HC in that study was defined using the PaCO₂ at the worst PaO₂ within 24 h, a very specific criterion for analysis. However, using discharge home as a surrogate for good neurological outcome [11] is questionable, as it may not directly reflect neurological recovery.

Consistent with this, a single-center study analyzing mean PaCO₂ found no significant association with CPC or survival [28]. Conversely, two single-center studies reported that HC, assessed either as any PaCO₂ ≥50 mmHg within the first 24 h after ROSC or as an initial PaCO₂ ≥50 mmHg after initiation of post-ROSC ventilator settings, was associated with worse CPC outcomes [29, 30]. Another multicenter study categorized patients into mild and severe HC groups, with mild HC showing better mRS and severe HC showing worse scores [31]. Focusing specifically on IHCA, a single-center study found that mild HC was not associated with neurological recovery by GOS, whereas severe HC was linked to worse GOS, and both mild and severe HC were associated with reduced survival, defined as GOS 4–5 [32].

Regarding survival outcomes, a multicenter study categorized mean PaCO₂ levels and found that mild HC was not associated with survival, whereas severe HC was linked to decreased survival [33]. In another large retrospective study incorporating both PaCO₂ and pH status, HC with normal pH was not associated with survival, while HC with hypercapnic acidosis was related to decreased survival [34].

Overall, these findings remain contradictory, similar to those in OHCA-only populations, despite broadly comparable baseline characteristics such as age and TTM use. The higher proportion of non-shockable rhythms likely reflects the inclusion of IHCA patients [27]. When the observational evidence across OHCA-only and mixed IHCA/OHCA populations is considered together, several patterns may help explain these heterogeneous findings. First, the PaCO₂ thresholds used to define HC varied across studies. Most studies used a threshold of >45 mmHg, whereas others used >50 mmHg. Several studies reporting favorable outcomes used the lower threshold. In studies that separated HC by severity, mild HC (generally defined as PaCO₂ 45–55 mmHg) was associated with favorable neurological outcomes in some studies and was not associated with worse neurological outcomes in others [21, 22, 26, 31–33]. In contrast, severe HC (generally defined as PaCO₂ >55 mmHg) was associated with worse neurological outcomes [22, 31–33]. These findings suggest a possible dose–response relationship. Second, the timing and method of PaCO₂ assessment differed across studies. Some studies used a single measurement, such as the first or highest PaCO₂ within 24 h [17, 20, 22–26], whereas others used mean or time-weighted values over 24–72 h [12, 18, 19, 21]. These methods capture different durations and burdens of hypercapnia and limit direct comparisons.

The interpretation of observational studies should account for reverse causality. Because most studies assessed HC as a prognostic marker rather than a deliberately induced intervention, observed HC may reflect underlying respiratory dysfunction, illness severity, or ventilatory management rather than a direct therapeutic effect. This concern is illustrated by differences in respiratory characteristics between outcome groups. Vaahersalo et al. [12] reported a lower prevalence of chronic respiratory disease among patients with favorable outcomes, whereas Kilgannon et al. [31] identified differences in ventilatory variables and acknowledged that abnormal PaCO₂ could reflect intrinsic patient characteristics, including more severe lung injury. Furthermore, the poorer survival associated with hypercapnic acidosis, compared with HC with normal pH [34], may reflect greater illness severity, brain injury severity, or residual confounding rather than a direct harmful effect of HC. Overall, observational associations cannot establish causality, highlighting the need for well-controlled interventional trials.

### Neurological and survival outcomes associated with hypercapnia in post-cardiac arrest patients: reports from special population models

In special population models, two studies from extracorporeal cardiopulmonary resuscitation (ECPR) cohorts have been published. A retrospective study measuring mean PaCO₂ over the first 72 h after cannulation found that HC (> 42 mmHg) was associated with worse CPC [35]. Conversely, a large retrospective study using PaCO₂ at 24 h, categorized as mild (45–54 mmHg) or severe (≥ 55 mmHg), found no association with composite acute brain injury (ischemic stroke, intracranial hemorrhage, seizure, or brain death) or survival [36]. Collectively, these retrospective findings do not establish a causal relationship between HC and neurological outcomes in the ECPR setting. Interpretation is limited by differences in the timing and assessment of PaCO₂ exposure, residual confounding, and ECPR-specific factors, including prolonged low-flow duration and ischemia–reperfusion injury. Therefore, the neurological effects of HC in this population remain uncertain and should not be directly extrapolated to conventional PCA models [37].

Interestingly, a multicenter study found that patients with COPD exposed to mild HC (46–55 mmHg) during the PCA phase of OHCA had better survival than those with NC, whereas non-COPD patients with elevated PaCO₂ at any level showed worse outcomes [38]. This observation aligns with the concept that COPD patients often live in a state of chronic hypercapnia to which their bodies have adapted [39]. Therefore, attempts to normalize PaCO₂ in this population may disrupt compensation, potentially increasing lung injury and reducing survival.

In pediatric populations, a multicenter study of IHCA patients assessing the highest PaCO₂ within 24 h and found that HC (> 50 mmHg) was associated with worse neurological outcomes, evaluated by pediatric CPC [40]. Consistently, other studies in children have shown that HC is associated with poorer survival [40, 41]. These findings may reflect physiological differences between children and adults, limited knowledge of cerebral autoregulation, and challenges in optimizing ventilatory management in pediatric care. Consequently, the mechanisms underlying these observations remain unclear. Given the limited and observational nature of the existing studies, stronger evidence is still required to define the role of HC in pediatric PCA care [42], as summarized in Table 2.

**Table 2:** Neurological and survival outcomes associated with hypercapnia in special post-cardiac arrest population

**Study type/** **reference**	**Population**	**TTM** **(**^**๐**^**C)**	**Number of participants**		**Hypercapnia**		**Major findings**		Interpretation*	**Key limitations**
							**Neurological outcome**	**Survival**		
			**HC**	**NC**	**Assessment definition**	**PaCO**_**2**_** level** **(mmHg)**				
Prospective, multicenter/ [40]	Pediatrics (term newborn – 18 y), IHCA	N/A	147	78	Highest PaCO₂ during the first 24 h after cardiac arrest	≥ 50	↓ favorable PCPC at hospital discharge	↓ at hospital discharge	In pediatric patients, hypercapnia was associated with lower survival rates and worse neurological outcomes	Intermittent and variably timed ABG measurements
Prospective, multicenter/ [41]	Pediatrics (1 mo – 18 y), IHCA	N/A	61	130	First PaCO₂ immediately after ROSC	> 50	N/A	↓ at hospital discharge	In pediatric patients, initial hypercapnia after ROSC was linked to lower survival rates at hospital discharge	Single PaCO₂ measurement at each time point
			25	118	PaCO₂ at 24 h after cardiac arrest	> 50	N/A	↔ at hospital discharge		
Retrospective, single-center/ [35]	Adult, ECPR, mixed IHCA& OHCA	Yes (N/A)	31	159	Mean PaCO₂ during the first 72 h after ECPR	> 42	↓ favorable CPC at hospital discharge	N/A	In the ECPR population, hypercapnia was linked to poor neurological outcomes	Exposure averaged over 72 h may obscure early timing and fluctuations; long study period with evolving post-arrest care
Retrospective, multicenter/ [36]	Adult, ECPR	N/A	Mild 321	1228	Single PaCO₂ measurement closest to 24 h after ECMO cannulation	Mild 45 – 54	↔ composite acute brain injury** within 24 h	↔ at hospital discharge	In the ECPR population, mild and severe hypercapnia were not associated with either composite acute brain injury or survival	exact exposure timing and duration unavailable; cardiac arrest and CPR characteristics incomplete
			Severe 58	1228		Severe ≥ 55	↔ Composite acute brain injury** within 24 h	↔ at hospital discharge		
Retrospective, multicenter/ [38]	Adult, COPD, OHCA	Yes (N/A)	N/A	N/A	Proportion of time spent in mild HC during the first 24 h after ICU admission	COPD with mild HC (46—55)	N/A	↑ at hospital discharge	Mild hypercapnia was associated with increased survival in COPD patients, but reduced survival in non-COPD patients	HC/NC group sizes not reported; PaCO₂ assessed after ICU admission; cardiac arrest and CPR characteristics unavailable; neurological outcomes not assessed
						COPD with severe HC (> 55)	N/A	↓ at hospital discharge		
						Non-COPD with mild HC (46—55)	N/A	↓ at hospital discharge		
						Non-COPD with severe HC (> 55)	N/A	↓ at hospital discharge		

*Interpretation compares findings in the hypercapnia group with the normocapnia group. ↑ indicates an increase or higher rate of the specified outcome; ↓ indicates a decrease or lower rate of the specified outcome; ↔ indicates no significant association or difference

**Composite acute brain injury included ischemic stroke, intracranial hemorrhage, seizure, and brain death

COPD, chronic obstructive pulmonary disease; CPC, Cerebral Performance Categories; ECPR, extracorporeal cardiopulmonary resuscitation; HC, hypercapnia; IHCA, in-hospital cardiac arrest; NC, normocapnia; OHCA, out-of-hospital cardiac arrest; PCPC, Pediatric Cerebral Performance Category scale; PaCO_2_, partial pressure of carbon dioxide in arterial blood; ROSC, return of spontaneous circulation; TTM, targeted temperature management.

In summary, ECPR cohorts showed no neurological or survival benefit from HC, reflecting the distinct physiology of extracorporeal support. In COPD patients, mild HC appeared favorable for survival, consistent with chronic adaptation, whereas non-COPD and pediatric IHCA patients demonstrated worse neurological outcomes and survival.

### Controlled trials with related sub-studies on clinical outcomes associated with hypercapnia in post-cardiac arrest adult patients

Although observational studies yielded inconsistent findings, several, particularly in OHCA populations [11, 12, 26], suggested that mild HC may be associated with favorable outcomes [26, 31], prompting subsequent controlled trials to test this hypothesis and explore related cardiovascular effects.

In the Carbon dioxide, Oxygen, and Mean arterial pressure After Cardiac Arrest and REsuscitation (COMACARE) pilot trial of OHCA patients with an initial shockable rhythm comparing high-normal (44–45 mmHg) and low-normal (34–35 mmHg) PaCO₂ during PCA care, regional cerebral oxygen saturation (rSO₂) measured by near-infrared spectroscopy (NIRS) was higher in the high-normal group. However, there were no significant differences in NSE, EEG grading, CPC at 6 months, 30-day survival, or myocardial injury as assessed by cardiac troponin T (TnT) [43]. This study found that rSO₂ values increased to levels comparable to those of healthy individuals in both groups [43, 44], although rSO₂ reflects only a limited frontal region and may not capture broader neurological recovery [45]. Consistent with these findings, a double cross-over study demonstrated that mild HC (50–55 mmHg) maintained for 30 min significantly increased rSO₂ compared with NC (35–45 mmHg), with values above normal ranges [44, 46]. These findings reflect short-term physiological effects.

The Carbon Control after Cardiac Arrest (CCC) trial [47], a multicenter randomized controlled trial (RCT), investigated therapeutic mild HC (50–55 mmHg for 24 h) compared with NC (35–45 mmHg) in both OHCA and IHCA patients. NSE levels were lower in the HC group following ROSC, suggesting reduced neuronal injury. However, there were no significant differences in S100B, ischemic changes on brain CT, extended GOS at 6 months, arrhythmia within 24 h, or survival. Importantly, the trial confirmed the feasibility and safety of maintaining mild HC, with no serious complications reported, forming the basis for a larger RCT to assess efficacy [47].

The Targeted Therapeutic Mild Hypercapnia after Resuscitated Cardiac Arrest (TAME) trial [48], the largest multicenter RCT to date on therapeutic HC after OHCA, compared 24 h of mild HC (50–55 mmHg) with NC (35–45 mmHg). The trial showed no improvement in long-term neurological outcomes (GOS-E, mRS, EQ-VAS) or 6-month survival [49]. Notably, controlled HC began a mean of 3 h after ROSC, suggesting that delayed initiation may have contributed to the null results. However, TAME enrolled only patients with OHCA and assessed 6-month patient-centered outcomes, whereas earlier studies included more heterogeneous populations and often focused on physiological measures, biomarkers, or shorter-term outcomes. These differences should be considered when comparing the findings. A TAME sub-study using sonographic assessment found that mild HC reduced cerebral microvascular resistance, consistent with vasodilation, without raising ICP, while MAP remained unchanged and CPP was unaffected [48]. These findings highlight only short-term physiological effects.

Interestingly, another TAME sub-study focused on acute myocardial infarction (AMI). In this group, mild HC was associated with lower cardiac TnT, reduced lactate, less resuscitation fluid, and hemodynamic improvements reflected by higher cardiac output, stroke volume, and cardiac index compared with NC, while no such effects were seen in non-AMI patients [50]. MAP remained unchanged [50]. Both groups developed mild respiratory acidosis, which was not associated with increased cardiac injury. Interpretation is limited by the use of cardiac TnT as a surrogate outcome, reflecting only short-term cardioprotective effects, and by delayed PaCO_2_ control post-cardiac intervention. Overall, mild HC reduced myocardial injury and improved hemodynamic performance in AMI patients, but did not improve survival [50].

In another TAME sub-study, pulmonary artery catheterization showed that mild HC was associated with improved cardiac output, stroke volume, and mixed venous oxygen saturation, together with lower systemic vascular resistance and improved fluid balance, while MAP remained unchanged [51]. Moreover, mild HC did not increase pulmonary vascular resistance or impair right ventricular function [51]. Of note, the HC group required more vasoactive–inotropic support, possibly due to reduced catecholamine responsiveness under mild respiratory acidosis [51, 52]. These findings suggest that mild HC was not linked to adverse cardiovascular effects and may transiently improve hemodynamic performance.

Another TAME sub-study provided a different perspective by testing the hypothesis that mild HC might mitigate acute kidney injury (AKI) related to cardiogenic shock after OHCA [53]. Although cardiogenic shock was strongly associated with AKI, there were no differences between HC and NC in AKI incidence or stage or need for renal replacement therapy. These findings suggest that mild HC did not prevent AKI, even in patients with cardiogenic shock [53], as summarized in Table 3.

**Table 3:** Controlled trials and related sub-studies of hypercapnia and clinical outcomes after adult cardiac arrest

**Models**	**Study type**	**TTM(**^**๐**^**C)**	**Number of participants**		**Intervention: Hypercapnia** **(mmHg)/Duration of PaCO₂ control**	**Major findings**					**Interpretation**	**Ref**
						**Neurological outcome**		**Survival**	**CVS**	**Others**		
			**HC**	**NC**		**Neurological Status**	**Serum biomarker**					
VF arrest, OHCA	RCTs, multi-center, COMACARE study	Yes (33 or 36)	59	61	44 – 45*/36 h	↔ CPC at 6 mo ↑ rSO_2_ during intervention by NIRS ↔ EEG grading at 48 h	↔ NSE ↔ S100B at 48 h	↔ at 1 mo	↔ cardiac TnT at 48 h	N/A	A high-normal PaCO_2_ levels increased cerebral oxygen saturation, without any effect on biomarkers of neurological change, cardiac injury or survival	[43]
IHCA& OHCA	Double cross over study, single-center	Yes (36)	7	7	50 – 55/30 min/period	↑ rSO_2_ by NIRS at 30 min	N/A	N/A	N/A	N/A	Mild hypercapnia increased cerebral oxygenation	[46]
IHCA& OHCA	RCTs, multi-center, The CCC Trial	Yes (N/A)	42	41	50 – 55/24 h	↔ GOS-E at 6 mo ↔ Ischemic findings on CT brain at 24 h	↓ NSE increase at 72 h ↔ S100B at 48 and 72 h	↔ at hospital discharge	↔ Arrhythmias at 24 h	N/A	Mild hypercapnia was associated with reduced NSE levels, without any correlation with survival rates, neurological outcomes, or cardiac effects	[47]
OHCA	RCTs, multi-center, TAME Trial	Yes (N/A)	829	839	50 – 55/24 h	↔ GOS-E ↔ mRS ↔ EQ Visual Analogue Scale at 6 mo	N/A	↔ at hospital discharge ↔ at 6 mo	N/A	N/A	Mild hypercapnia did not give favorable neurological outcomes or survival	[49]
OHCA	Sub-study TAME, single-center	Yes (N/A)	7	5	50 – 55/24 h	↓ CMVR at MCA by U/S ↔ ICP by U/S at 24 h	N/A	N/A	↔ MAP at 24 h	N/A	Mild hypercapnia decreased cerebral microvascular resistance without increased intracranial pressure	[48]
OHCA	Sub-study TAME, single-center	Yes (33)	31 (with AMI)	26 (with AMI)	50 – 55/24 h	N/A	N/A	↔ at hospital discharge ↔ at 6 mo	↓ Cardiac TnT ↓ Lactate ↑ CPO ↑ SV ↑ SvO_2_ ↑ CI ↓ SVRI ↔ MAP ↔ HR ↔ UOP ↓ Resuscitation fluids ↔ Vasoactive-inotropic use at 24, 48, and 72 h	↑ Mild respiratory acidosis	In AMI patients, mild hypercapnia was associated with reduced myocardial injury and improved cardiac performance	[50]
			31 (without AMI)	37 (without AMI)	50 – 55/24 h	N/A	N/A	N/A	↔ Cardiac TnT ↔ Lactate ↑ SvO_2_ ↑ CI ↔ CPO ↔ SV ↔ HR ↔ UOP ↔ Resuscitation fluids ↑ Vasoactive-inotropic use at 24, 48, and 72 h	↑ Mild respiratory acidosis		
OHCA	Sub-study TAME, single-center	Yes (33)	41	43	50 – 55/24 h	↔ GOS-E at 6 mo	N/A	N/A	↑ SvO_2_ ↑ CI ↑ SV ↑ CPO ↑ mPAP ↓ SVRI ↔ PVRI ↔ PAPI ↔ RAP ↔ MAP ↔ HR ↔ PCWP ↔ PaO_2_/FiO_2_ ratio ↓ Resuscitation fluids ↔ PCI success rates ↑ Doses of NE at 24 h	↑ Mild respiratory acidosis at 24 h	Mild hypercapnia was associated with improved cardiac performance and mixed venous oxygen saturation	[51]
OHCA	Sub-study TAME, multi-center	Yes (N/A)	476 (with AKI + CS)	460 (with AKI + CS)	50 – 55/24 h	↔ GOS-E at 6 mo	N/A	↔ at 6 mo	N/A	↔ worst AKI ↔ RRT at 7 days	There was no association between mild hypercapnia and worsening of AKI or the need for RRT, either with or without cardiogenic shock	[53]
			120 (with AKI + no-CS)	147 (with AKI + no-CS)	50 – 55/24 h	↔ GOS-E at 6 mo	N/A	↔ at 6 mo	N/A	↔ worst AKI ↔ RRT at 7 days		

*Values originally reported in kPa were converted to approximate values in mmHg for consistency of presentation

AKI, acute kidney injury; AMI, acute myocardial infarction; CI, cardiac index; CMVR, cerebral microvascular resistance; CPC, Cerebral Performance Categories scale; CPO, cardiac power output; CS, cardiogenic shock; CVS, cardiovascular system; EEG, electroencephalography; GOS-E, Glasgow Outcome Scale–Extended; HC, hypercapnia; HR, heart rate; ICP, intracranial pressure; IHCA, in-hospital cardiac arrest; MAP, mean arterial pressure; MCA, middle cerebral artery; mPAP, mean pulmonary artery pressure; mRS, Modified Rankin Score; NC, normocapnia; NE, norepinephrine; NIRS, near-infrared spectroscopy; NSE, serum concentration of neuron-specific enolase; OHCA, out-of-hospital cardiac arrest; PaCO₂, partial pressure of carbon dioxide in arterial blood; PaO₂/FiO₂, ratio of arterial oxygen partial pressure to fractional inspired oxygen; PAPI, pulmonary artery pulsatility index; PCI, percutaneous coronary intervention; PCWP, pulmonary capillary wedge pressure; PVRI, pulmonary vascular resistance index; RAP, right atrial pressure; RCT, randomized controlled trial; RRT, renal replacement therapy; rSO₂, regional cerebral oxygen saturation; S100B, S100 calcium-binding protein B; SV, stroke volume; SVRI, systemic vascular resistance index; TnT, troponin T; TTM, Targeted Temperature Management; UOP, urine output; U/S, ultrasound; VF, ventricular fibrillation.

Several contemporary systematic reviews and meta-analyses have shown that mild HC is not associated with worse neurological or survival outcomes and does not improve long-term prognosis [54, 55]. Some evidence suggests better short-term neurological outcomes [56], whereas PaCO₂ levels above the mild HC range have been linked to adverse effects [57]. Overall, mild HC appears safe, yet its clinical benefit remains uncertain [54–57].

Taken together, controlled trials and meta-analyses demonstrate that maintaining mild HC in PCA care is feasible and safe, enhancing short-term physiological markers of cerebral oxygenation and neuronal injury. However, these effects have not translated into consistent improvements in functional outcomes. Sub-studies suggest possible short-term hemodynamic benefits and reduced cardiac injury biomarkers, but without clear clinical impact, and survival remains unchanged.

### Impact of hypercapnia on post-cardiac arrest outcomes: evidence from in vivo models

Three in-vivo studies investigating mild HC during the PCA period were conducted to explore underlying mechanisms, inspired by the CCC trial. In one study, pigs were induced to asphyxial cardiac arrest for 4 min before CPR [58]. After ROSC, CO₂ was regulated for 12 h using end-tidal carbon dioxide (EtCO₂) guidance to maintain either mild HC (45–50 mmHg) or NC (35–40 mmHg) [58]. Mild HC increased brain tissue oxygen tension (PbtO₂) to levels comparable to those before arrest without raising arterial oxygenation [58, 59]. It was accompanied by higher MAP and ICP, but CPP was not changed. There were no group differences in diffusion limitation (jugular bulb–PbtO₂ gradient), NSE, S100β, cardiac output, extravascular lung water, or survival [58]. These findings suggest that mild HC improves short-term cerebral oxygenation without evidence of neurological benefit.

In another study representing an OHCA model due to myocardial infarction, ventricular fibrillation (VF) arrest was induced in pigs by left anterior descending coronary occlusion, followed by a prolonged no-flow period before CPR and delayed defibrillation [60]. After ROSC, ventilation was adjusted to maintain mild HC (EtCO₂ 45–50 mmHg) or NC (35–40 mmHg) for 4 h [60]. Mild HC increased MAP and reduced neuronal degeneration in the frontal cortex. Although ICP was not measured, the absence of vascular leakage on albumin staining indicated no brain edema. However, there were no differences in hippocampal injury, NSE, cardiac TnT, myocardial infarct size, cardiac performance, or survival [60]. These findings suggest that mild HC increases arterial pressure and reduces neuronal degeneration in specific cortical regions, but does not confer benefits in other outcomes.

The latest study incorporating cardiopulmonary bypass (CPB) resuscitation and TTM assessed mild HC in PCA care [61]. VF was induced in pigs and left untreated for 10 min before CPB was initiated to achieve ROSC. After 30 min of stabilization, PaCO₂ was controlled for two hours to mild HC (6.5–7.0 kPa; approximately 49–53 mmHg) or NC (5.0–5.5 kPa; approximately 38–41 mmHg), with or without TTM at 33 °C. Mild HC increased CBF in both TTM and non-TTM groups and, when combined with TTM, reduced brain lactate, pyruvate, lactate/pyruvate ratio, and glycerol measured by cerebral microdialysis, while maintaining stable ICP and CPP, indicating lower metabolic demand, preserved aerobic metabolism, and possible neuroprotection. In contrast, without TTM, mild HC was associated with increased ICP, decreased CPP, and elevated glutamate, suggesting neuronal stress. MAP and cardiac output were unchanged with TTM, but decreased without TTM. However, mild HC was maintained for only two hours, and the study did not directly compare the groups with and without TTM, making it difficult to conclude whether the observed improvement was due to a synergistic effect of HC and TTM or to TTM alone [61], as summarized in Table 4.

**Table 4:** Impact of hypercapnia on outcomes in in vivo post-cardiac arrest models

**Species**	**Models of cardiac arrest**	**CPR methods**	**TTM** **(**^๐^C**)**	**Intervention: Hypercapnia** **(mmHg)/Duration of PaCO₂ control**	**Major findings**					**Interpretation**	**Ref**
					**Neurological outcomes**			**Survival**	**CVS**		
					**Favorable neurological recovery**	**Brain histology, physiology, and metabolism**	**Serum biomarker**				
Male Bama miniature pigs	Asphyxia by ET-tube clamping	Manual CPR	No	45–50*/12 h	N/A	↑ PbtO_2_ ↔ CPP ↔ Δ (PjvO₂—Pbto₂) ↔ Temperature ↑ ICP at 12 h	↔ NSE ↔ S100β ↔ LOI at 12 h	↔ at 12 h	↑ MAP ↔ CO ↔ EVLW ↔ AJDo_2_ ↔ Δ(PjvCO₂—PaCO₂) ↓ PaO_2_ at 12 h	In an asphyxial cardiac arrest model, mild hypercapnia improved brain tissue oxygen tension and mean arterial pressure. However, it increased intracranial pressure	[58]
Male domestic pigs	VF arrest by LAD occlusion and/or 1–2 mA AC	Mechanical CPR	No	45–50*/4 h	↔ OPC at 96 h ↔ NAS, NDS at 24, 48, and 72 h	↓ ND (frontal cortex) ↔ ND (hippocampus) ↔ Apoptotic neurons (caspase−3) ↔ IVL (albumin staining) at 96 h	↔ NSE at 96 h	↔ at 96 h	↑ MAP ↑ SAP ↑ DAP ↔ HR ↔ LVEF ↔ SV at 2,4 and 96 h ↔ Cardiac TnT ↔ pH in ABG at 2, 4, and 96 h ↔ Myocardial infarct (histology) at 96 h	In a VF cardiac arrest model, mild hypercapnia improved arterial pressure and reduced neuronal degeneration but did not improve survival	[60]
Both sexes crossbreed Norwegian Landrace pigs	VF arrest by 90V AC	Cardio-pulmonary bypass	No	49–53^†^/2 h	N/A	↑ CBF ↑ Temperature ↑ PtCO_2_ ↑ ICP ↑ Glutamate ↔ CaF ↔ PRx ↔ Lactate ↔ LP ratio ↔ Glycerol ↓ CPP ↓ Pyruvate ↓ Glucose at 2 h	N/A	N/A	↓ MAP ↓ CO at 2 h	In a VF cardiac arrest model, mild hypercapnia had neuroprotective effects during TTM but caused adverse hemodynamic effects without TTM	[61]
			Yes (33)	49–53^†^/2 h	N/A	↑ CBF ↑ Temperature ↑ PtCO_2_ ↔ ICP ↔ CPP ↔ PRx ↔ CaF ↔ Glutamate ↓ Lactate ↓ Pyruvate ↓ Glycerol ↓ LP ratio ↓ Glucose at 2 h	N/A	N/A	↔ MAP, CO at 2 h		

ABG, arterial blood gas; AC, alternating current; AJDo₂, arteriojugular oxygen difference; CaF, cerebral autoregulation function; CBF, cerebral blood flow; CO, cardiac output; CPP, cerebral perfusion pressure; CPR, cardiopulmonary resuscitation; CVS, cardiovascular system; DAP, diastolic arterial pressure; ET, endotracheal tube; EVLW, extravascular lung water; HR, heart rate; ICP, intracranial pressure; IVL, interstitial vascular leakage; LAD, left anterior descending coronary artery; LOI, lactate oxygen index; LP ratio, lactate/pyruvate ratio; LVEF, left ventricular ejection fraction; MAP, mean arterial pressure; NAS, neurological alertness score; ND, neuronal degeneration; NDS, neurological deficit score; NSE, serum concentration of neuron-specific enolase; OPC, overall performance category; PaCO₂, partial pressure of carbon dioxide in arterial blood; PaO₂, partial pressure of oxygen in arterial blood; PbtO₂, brain tissue oxygen tension; PRx, pressure reactivity index; PtCO₂, brain tissue carbon dioxide partial pressure; S100B, S100 calcium-binding protein B; SAP, systolic arterial pressure; SV, stroke volume; TnT, troponin T; TTM, Targeted Temperature Management; VF, ventricular fibrillation; Δ(PjvCO₂ – PaCO₂), difference in partial pressure of venous and arterial CO₂; Δ(PjvO₂ – PbtO₂), difference in partial pressure of venous and brain tissue O₂.

*This study controlled hypercapnia and normocapnia using the end-tidal CO₂ method.

^**†**^Values originally reported in kPa were converted to approximate values in mmHg for consistency of presentation.

Viewed across these in vivo experiments, the findings were consistent with clinical studies. Mild HC improved short-term neurological parameters, most evident in invasive monitoring studies showing increased PbtO₂ [58], favorable cerebral microdialysis profiles [61], and reduced cortical neuronal injury in the frontal cortex [60]. However, hippocampal CA1 injury remained unaffected, indicating region-specific effects of mild HC [60, 62]. Hemodynamic and metabolic responses also varied with TTM, suggesting that therapeutic hypothermia may modulate the cerebral effects of mild HC [61]. Animal models failed to show the reduced myocardial injury observed in clinical sub-studies, making the cardiac effects of mild HC uncertain [50, 60]. These data extend clinical observations but do not clarify underlying mechanisms.

### Exploring therapeutic hypercapnia in post-cardiac arrest care: still promising or time to move on?

**Fig. 1:**
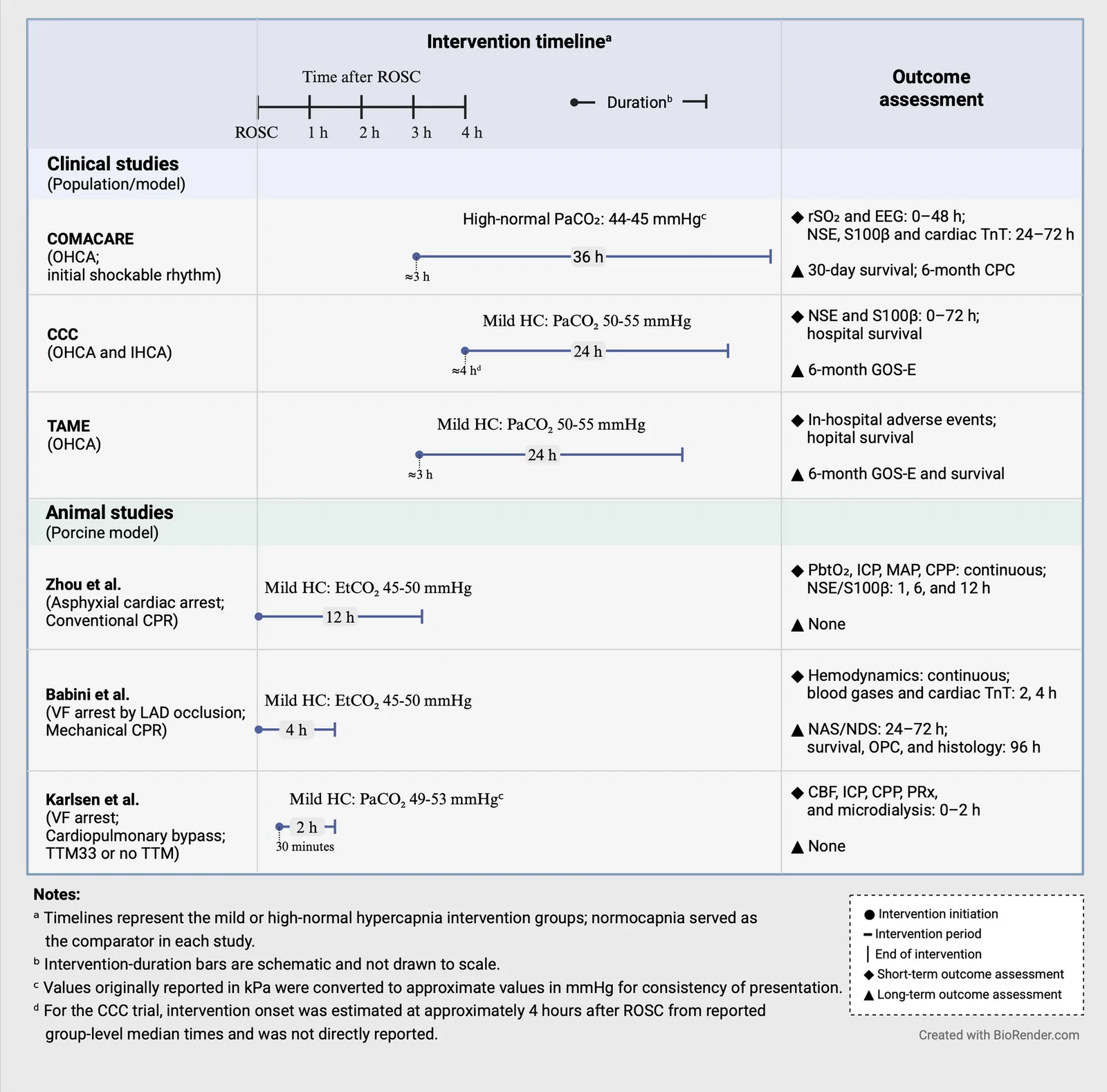
Timeline of intervention initiation, duration, and outcome assessment in clinical and animal studies of mild hypercapnia after cardiac arrest. Timelines represent the mild or high-normal hypercapnia intervention groups, with normocapnia serving as the comparator in each study. Solid circles indicate intervention initiation, horizontal bars indicate intervention duration, vertical ticks indicate intervention completion, diamonds indicate short-term outcome assessments, and triangles indicate long-term outcome assessments. Intervention initiation is positioned relative to return of spontaneous circulation (ROSC), whereas intervention-duration bars are schematic and not drawn to scale. For the CCC trial, intervention onset was estimated at approximately 4 h after ROSC from reported group-level median times and was not directly reported. Values originally reported in kilopascals were converted to approximate values in millimeters of mercury for consistency of presentation. CBF, cerebral blood flow; CCC, Carbon Control after Cardiac Arrest; COMACARE, Carbon dioxide, Oxygen, and Mean arterial pressure After Cardiac Arrest and Resuscitation; CPC, Cerebral Performance Category; CPP, cerebral perfusion pressure; CPR, cardiopulmonary resuscitation; EEG, electroencephalography; EtCO₂, end-tidal carbon dioxide; GOS-E, Glasgow Outcome Scale–Extended; HC, hypercapnia; ICP, intracranial pressure; IHCA, in-hospital cardiac arrest; MAP, mean arterial pressure; NAS, neurological alertness score; NDS, neurological deficit score; NSE, neuron-specific enolase; OHCA, out-of-hospital cardiac arrest; OPC, overall performance category; PaCO₂, partial pressure of carbon dioxide in arterial blood; PbtO₂, brain tissue oxygen tension; PRx, pressure reactivity index; ROSC, return of spontaneous circulation; rSO₂, regional cerebral oxygen saturation; S100B, S100 calcium-binding protein B; TAME, Targeted Therapeutic Mild Hypercapnia After Resuscitated Cardiac Arrest; TnT, troponin T; TTM, Targeted Temperature Management; VF, ventricular fibrillation

Mild HC (PaCO₂ 50–55 mmHg) has been used in both clinical and experimental settings [46, 47, 49, 61]. Some protocols targeted EtCO₂ 45–50 mmHg, resulting in comparable PaCO₂ levels [58, 60]. This target range is also consistent with observational evidence suggesting that more severe HC (generally defined as PaCO₂ >55 mmHg) may be associated with poorer clinical outcomes [22, 31–33]. Mild HC can be implemented through ventilator adjustment. However, intervention protocols varied substantially across studies. The duration of HC ranged from 30 min [46] to 36 h [43], and the optimal treatment duration remains unknown. Current guidelines recommend targeting normocapnia during initial stabilization after sustained ROSC [4, 5]. In practice, CO₂ control in clinical trials was generally initiated approximately 2–5 h after ROSC [43, 47, 49]. Post-cardiac arrest brain injury evolves through time-dependent phases, and interventions targeting reperfusion–repolarization injury mechanisms may have a narrow therapeutic window [63]. If the potential neuroprotective effects of mild HC are greatest during this early phase, this delayed initiation may have attenuated any effect on long-term neurological outcomes. In contrast, animal studies initiated hypercapnia immediately after ROSC and reported favorable short-term physiological effects [58, 60], as illustrated in Fig. 1.

Neurologically, mild HC increases cerebral oxygenation during intervention, as evidenced by elevated rSO₂ [43, 46] and higher PbtO₂ [58]. Cerebral microdialysis suggests lower metabolic demand and reduced frontal cortical neuronal injury [60, 61], indicating region-specific neuroprotection. However, hippocampal injury, typically more sensitive to hypoxia, remained unchanged [60, 62]. NSE and S100B showed inconsistent results [43, 47, 58, 60], and no improvement was observed in long-term neurological outcomes [43, 47, 49, 60]. Overall, mild HC appears to enhance short-term cerebral physiology without translating into sustained neurological recovery.

Mechanistically, mild HC may increase CBF by reducing cerebrovascular resistance (CVR) through vasodilation, expressed as (CBF = CPP / CVR), where (CPP = MAP – ICP). Across studies, mild HC was associated with higher CBF [61] and with lower cerebral microvascular resistance, which is consistent with reduced downstream CVR [48]. CPP remained stable because ICP and MAP tended to change in the same direction [48, 58, 61]. Mild HC may support cerebral oxygenation via increased CBF without major complications such as brain edema [60]. Moreover, concerns that cerebral autoregulation may be impaired after cardiac arrest are partly mitigated by evidence showing preserved CPP and a maintained cerebrovascular response to PaCO₂ changes [9].

Mild HC was associated with reduced cardiac TnT and improved cardiac performance, including higher cardiac output and stroke volume, particularly in the AMI group, in TAME sub-studies [50, 51]. In contrast, animal models showed no difference in troponin T, histological myocardial injury, or hemodynamic parameters [58, 60]. Differences in experimental models and the timing of intervention may partly explain why the cardiac effects and underlying mechanisms of mild HC remain uncertain. Supporting this**,** an *ex-vivo* porcine perfusion study also found that short-term HC enhanced coronary flow, indicating a possible CO₂-mediated microvascular effect [64].

**Fig. 2:**
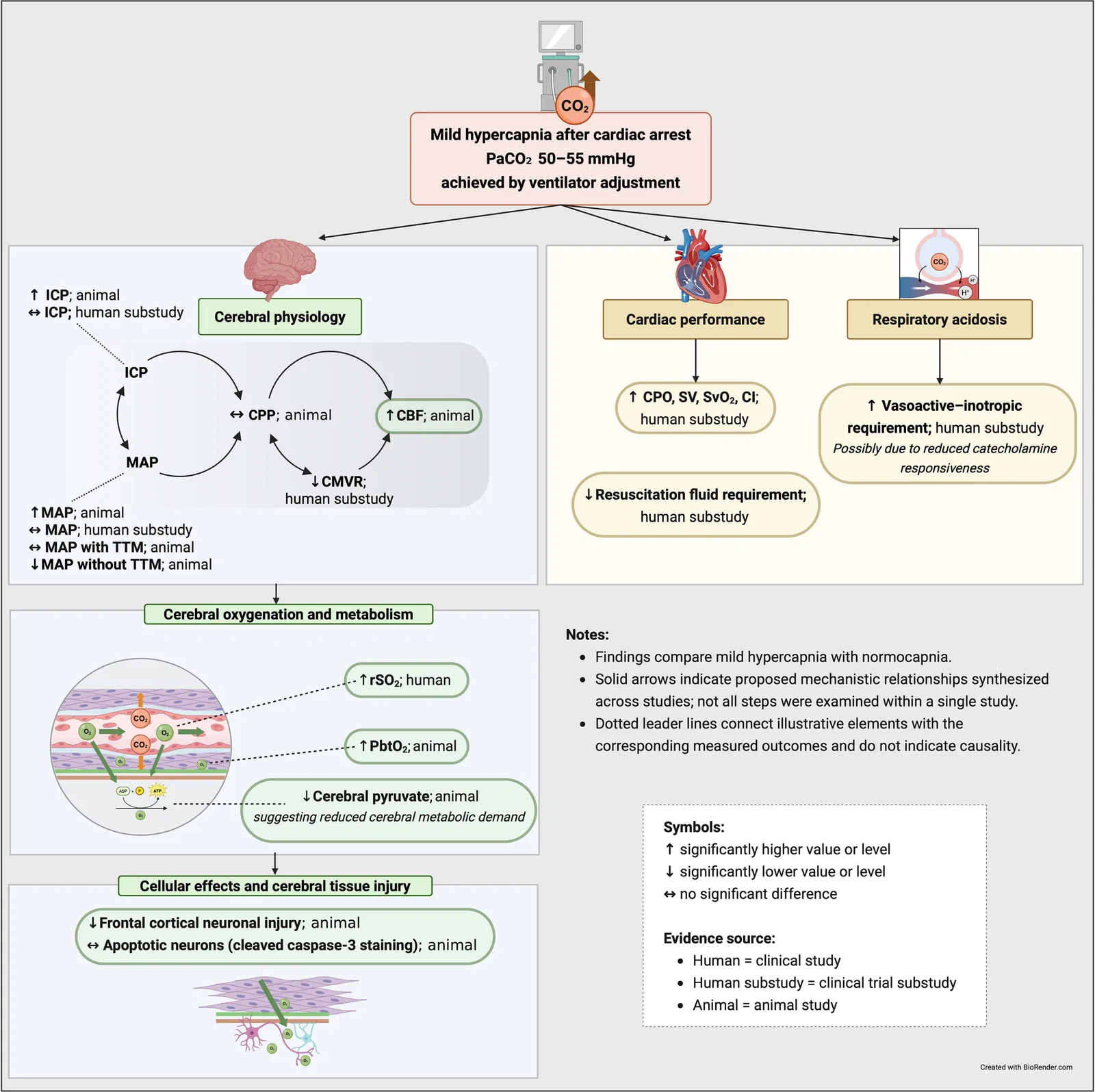
Mechanistic effects of mild hypercapnia after cardiac arrest. Mild hypercapnia, targeting a PaCO₂ of 50–55 mmHg through ventilator adjustment, may influence cerebral physiology, cerebral oxygenation and metabolism, cellular and cerebral tissue injury, and cardiovascular function. Cerebral effects include changes in ICP and MAP with preserved CPP, increased CBF, increased rSO₂ and PbtO₂, and reduced cerebral pyruvate, suggesting reduced cerebral metabolic demand. Animal studies also suggest reduced frontal cortical neuronal injury, whereas apoptotic neurons assessed by cleaved caspase−3 staining were unchanged. Cardiovascular findings from human substudies include increased CPO, SV, SvO₂, and CI, reduced resuscitation fluid requirements, and increased vasoactive–inotropic requirements, the latter possibly related to reduced catecholamine responsiveness during respiratory acidosis. Findings are presented relative to normocapnia. Solid arrows indicate proposed mechanistic relationships synthesized across studies; not all steps were examined within a single study. Dotted leader lines connect illustrative elements with the corresponding measured outcomes and do not indicate causality. **Symbols**: ↑, significantly higher value or level; ↓, significantly lower value or level; ↔, no significant difference. **Evidence sources**: Human, clinical study; human substudy, clinical trial substudy; animal, animal study. CBF, cerebral blood flow; CI, cardiac index; CMVR, cerebral microvascular resistance; CPO, cardiac power output; CPP, cerebral perfusion pressure; ICP, intracranial pressure; MAP, mean arterial pressure; PaCO₂, partial pressure of arterial carbon dioxide; PbtO₂, brain tissue oxygen tension; rSO₂, regional cerebral oxygen saturation; SV, stroke volume; SvO₂, mixed venous oxygen saturation; TTM, targeted temperature management

Adverse effects of mild HC in PCA care warrant consideration. Mild respiratory acidosis was associated with increased vasoactive–inotropic requirements, possibly due to reduced catecholamine responsiveness during acidosis [50, 51, 65]. However, acidosis severity did not affect survival, contrasting with some observational studies [34]. No major adverse effects of mild HC were observed, including arrhythmia [47], cardiogenic shock [53], lung injury [51, 58], kidney injury [53], or cerebral edema. Overall, neither controlled trials nor animal studies have demonstrated differences in survival between mild HC and NC [43, 47, 49–51, 53, 58, 60]. The proposed cerebral, cardiovascular, and cellular effects of mild HC, together with the supporting evidence from human and animal studies, are summarized in Fig. 2.

In summary, mild HC is still promising and warrants further investigation for its potential effects on PCA outcomes. Although it is a simple and safe intervention associated with improved cerebral oxygenation and possible cardioprotective effects, current evidence remains insufficient to support its use as a therapeutic strategy. Nevertheless, these findings broaden understanding of PaCO₂ management beyond the conventional normocapnic range (PaCO₂ 35–45 mmHg) and may inform its integration into individualized PCA care, such as in patients receiving lung-protective ventilation [66, 67] or those with chronic HC, such as COPD [38].

## Conclusions

Mild HC can improve short-term neurological outcomes in PCA care, but evidence for long-term neurological, cardiovascular, and survival benefits remains limited and inconclusive. At present, its impact is insufficient to justify adoption as a standalone therapeutic intervention. Further research should define its optimal role within PCA management and clarify underlying mechanisms.

### Future research perspectives

Future studies should clarify the role of mild HC in PCA care, particularly regarding the optimal timing of intervention, as existing clinical trials have typically initiated the intervention several hours after ROSC, potentially missing an early therapeutic window. Animal studies examining different intervention timings after ROSC are needed to test this hypothesis. The interaction with TTM also requires attention, given conflicting findings and the possibility that mild HC during hypothermia may better maintain cerebral oxygenation than strict normocapnia. In addition, future work should examine potential synergistic and adverse effects of increasing cerebral oxygenation without supplemental oxygen, such as reperfusion-related injury. Further controlled studies in patients with chronic HC, such as those with COPD, and mechanistic studies exploring its effects on the central nervous, cardiovascular, and inflammatory systems are needed to refine its potential role within integrated PCA management.

### Take-home message

Therapeutic mild hypercapnia after cardiac arrest improves short-term cerebral oxygenation without a clear major safety signal, but durable neurological or survival benefits remain unproven. Future studies should clarify the mechanisms linking these physiological effects to clinical outcomes and determine whether earlier initiation and optimized dose and duration can yield clinically meaningful benefits.

### Tweet

Mild hypercapnia improves cerebral oxygenation after cardiac arrest, but clinical benefits remain unproven. More investigations are needed.

## Data Availability

Data sharing is not applicable to this article as no datasets were generated or analyzed.

## Declarations

### Generative AI in scientific writing

During the preparation of this work, the authors used ChatGPT (OpenAI, San Francisco, CA, USA) to improve readability and language. The authors reviewed and edited the content as needed and take full responsibility for the final manuscript.
